# Supplementary material for: Myopathy associated LDB3 mutation causes Z-disc disassembly and protein aggregation through PKCα and TSC2-mTOR downregulation
Source: Commun Biol. 2021 Mar 19;4:355. doi: 10.1038/s42003-021-01864-1 (PMC7979776; doi:10.1038/s42003-021-01864-1)
Supplement: Supplementary file 2 — Supplementary Information [file 42003_2021_1864_MOESM2_ESM.pdf]

Supplementary Figures and Tables

Myopathy associated LDB3 mutation causes Z-disc disassembly and protein aggregation through PKCα and TSC2-mTOR downregulation

Pankaj Pathak[1#], Yotam Blech-Hermoni[1#], Kalpana Subedi[1], Jessica Mpamugo[1], Charissa Obeng-Nyarko[1], Rachel Ohman[1], Ilda Molloy[1], Malcolm Kates[1], Jessica Hale[1], Stacey Stauffer[2], Shyam K. Sharan[2], Ami Mankodi[1*]

[1]Neurogenetics Branch, National Institute of Neurological Disorders and Stroke, Bethesda, Maryland and [2]Mouse Cancer Genetics Program, Center for Cancer Research, National Cancer Institute, Frederick, MD

[#]These authors contributed equally.

*Corresponding author contact information: Ami Mankodi, MD, 35 Convent Drive, Building 35, Room 2A-1002, Bethesda, MD 20892-3075. Phone: (301) 827-6690, Fax: (301) 480-3365, Ami.Mankodi@nih.gov

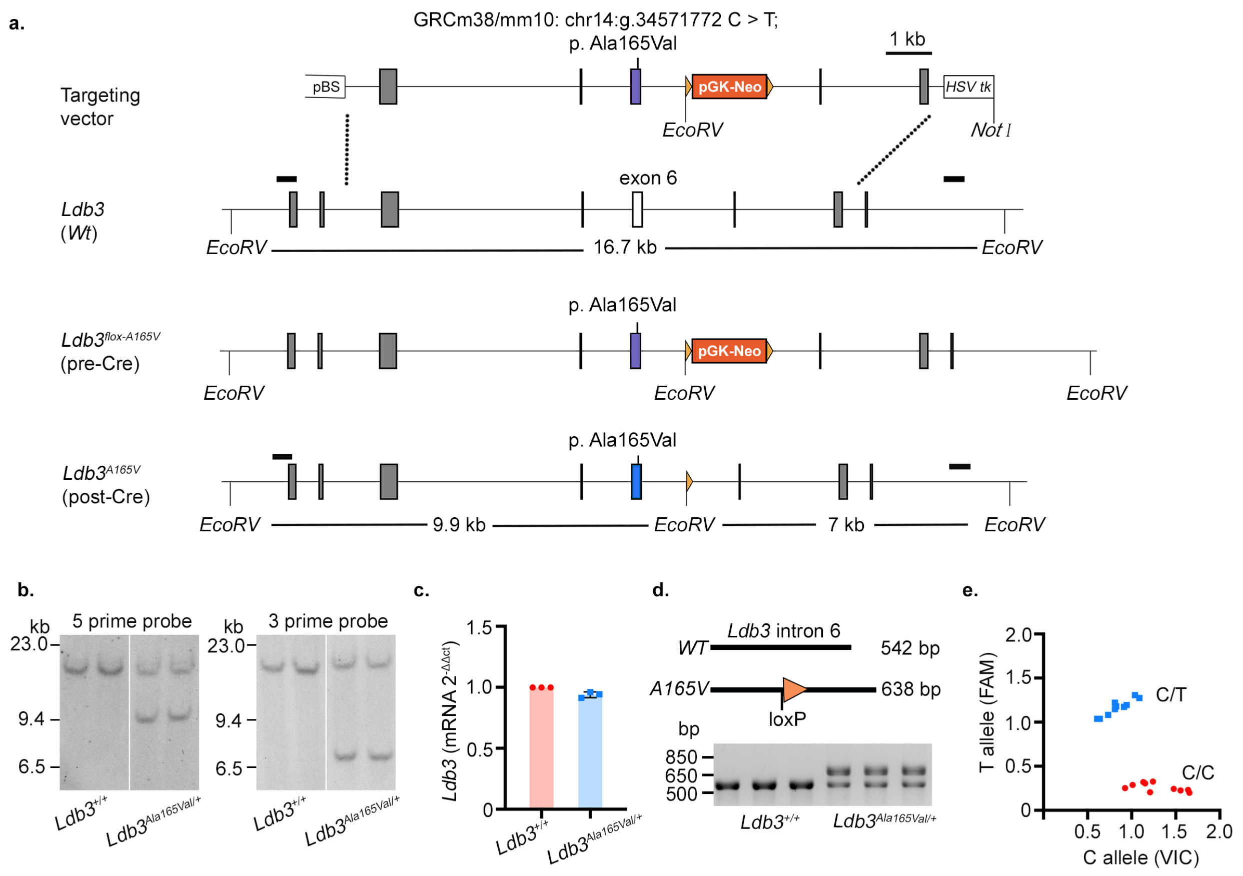

Supplementary Fig. 1: Generation of *Ldb3*$^{Ala165Val/+}$ knock in mice using recombineering technique. a, Gene targeting by homologous recombination to generate the *Ldb3*^{Ala165Val} knock in allele. Top panel shows the targeting vector containing introns 3 through 8 region of the mouse *Ldb3* gene (dotted lines). Boxes represent the exons 4 through 8. Single base C was changed to T at position chr14:g.3451772 (GRCm38/mm10) resulting in the exon 6 p.Ala165Val mutation (purple) and the neo cassette was inserted into intron 6. A wild type *Ldb3* allele (*Wt*) is shown as reference. The neo selection cassette flanked by loxP sites (orange triangles) in the *Ldb3*$^{flox\text{-} Ala165Val}$ allele was removed by breeding with *b-actin-Cre* mice resulting in activation of the *Ldb3*^{Ala165Val} allele (exon 6 in blue). Scale bar represents 1 kb.

Locations of Not I and EcoR V sites as well as 5' and 3' probes (black bars) for Southern blotting are shown. **b,** Representative Southern blot analysis of genomic DNA of *Ldb3*$^{Ala165Val/+}$ mice and *Ldb3*$^{+/+}$ littermates is shown. Mouse genomic DNA was digested by EcoR V and then separated on a 0.7% agarose gel. The *Wt* allele gave a band of 16.7 kb, whereas the *Ldb3*^{Ala165Val} allele gave 9.9 kb and 7.0 kb bands for the 5' and 3' probes, respectively. **c,** Bar dot plot of real time RT-qPCR data of *Ldb3* mRNA expression in the vastus muscle of 4 month old *Ldb3*$^{Ala165Val/+}$ mice (blue) normalized to *Ldb3*$^{+/+}$ littermates (red). The relative *Ldb3* mRNA levels were determined against the *Myom1* mRNA level. Data represent mean ± SD, n = 3 mice in triplicate assay. **d**, Representative gel shows that genotyping PCR using primers flanking the residual loxP site (orange triangle) in intron 6 generates a 638bp band from the *Ldb3*^{Ala165Val} allele and a 542 bp band from *Wt* allele. **e**, Representative allelic discrimination plot shows distinct clusters of *Ldb3*$^{+/+}$ (C/C; red; n = 10 mice) and *Ldb3*$^{Ala165Val/+}$ heterozygote (C/T; blue; n = 10 mice).

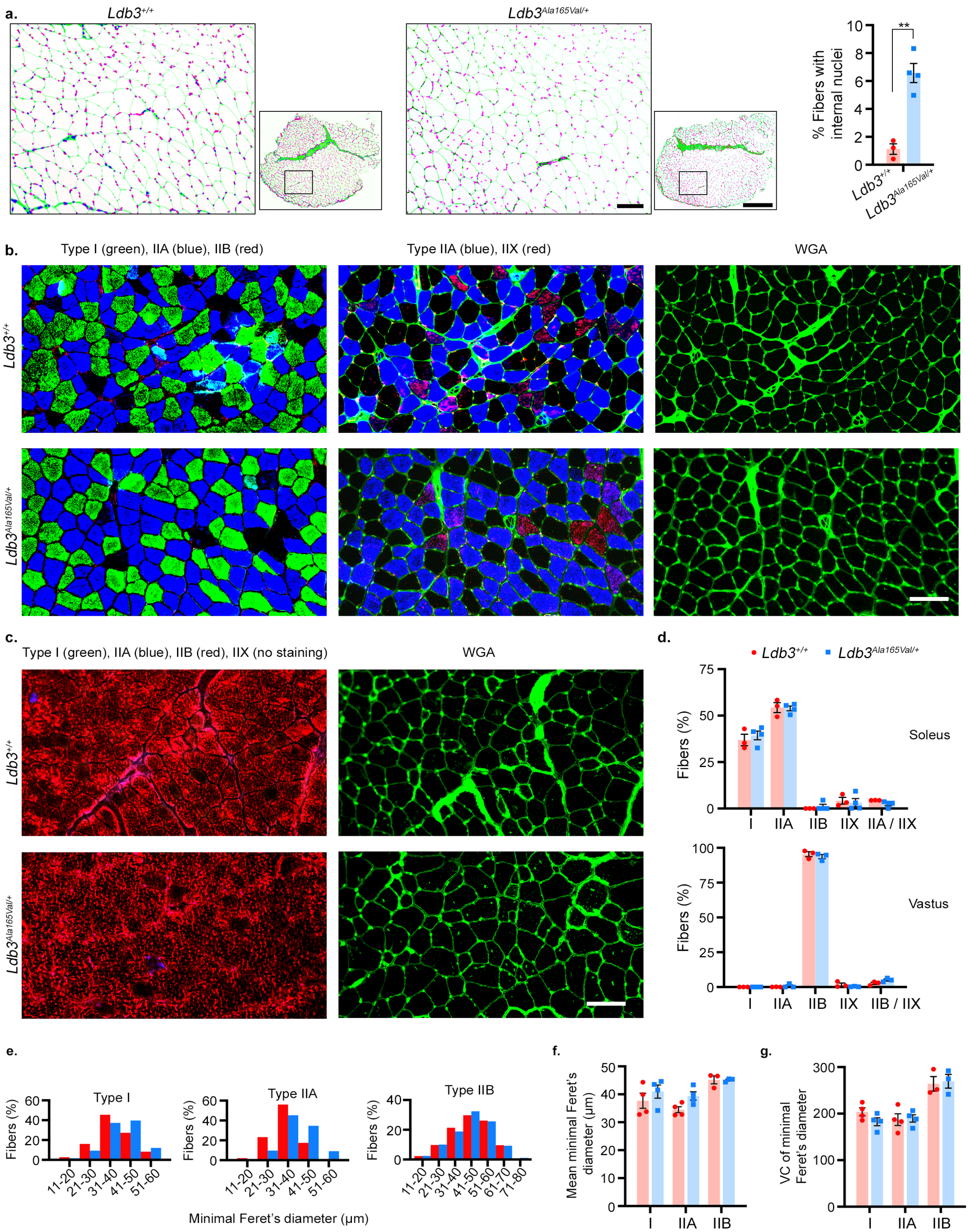

Supplementary Fig. 2: Quantitation of percentage internal nuclei, fiber type, and fiber-type specific size in skeletal muscle of *Ldb3*$^{Ala165Val/+}$ mice.

a, Immunofluorescence of the tibialis anterior muscle transverse section of 8 month old *Ldb3*$^{Ala165Val/+}$ mice (n = 4) and *Ldb3*$^{+/+}$ mice (n = 3) labeled with wheat germ agglutinin (WGA; muscle membrane; green) and DAPI (nuclei; magenta). The rectangles in insets define the boundary of main images. Percent fibers with internal nuclei in *Ldb3*$^{Ala165Val/+}$ mice (blue; Mean ± SEM: 6.6% ± 0.7%) and *Ldb3*$^{+/+}$ mice (red; Mean ± SEM: 1.1% ± 0.4%) are shown in bar-dot plot representing a mean of 764 fibers per muscle (Unpaired two-tailed *t-test* $p < 0.01$). **b-c,** Immunofluorescence of the soleus (b) and vastus (c) muscle transverse section of 8 month old *Ldb3*$^{Ala165Val/+}$ mice (n = 4 and 3, respectively) and *Ldb3*$^{+/+}$ mice (n = 3 each) labeled with myosin heavy chain types I, IIA, IIB, and IIX antibodies for fiber typing and WGA for muscle fiber size (minimal Feret's diameter) measurement. **d,** Fiber type percentages are shown for *Ldb3*$^{Ala165Val/+}$ mice (blue) and *Ldb3*$^{+/+}$ mice (red) in bar-dot plots. The data represent a mean of 615 fibers and 436 fibers per muscle, in the soleus and vastus muscles, respectively. **e,** Histogram of the fiber-type specific size in *Ldb3*$^{Ala165Val/+}$ mice (blue) and *Ldb3*$^{+/+}$ mice (red) are shown. The data represent a mean of 100 type I fibers, 141 type IIA fibers, and 288 type IIB fibers per muscle, and n = 4 mice for types I and IIA fibers and n = 3 mice for type IIB fibers. **f,** The mean minimal Feret's diameter of fiber types in *Ldb3*$^{Ala165Val/+}$ mice (blue) and *Ldb3*$^{+/+}$ mice (red) are shown in bar-dot plot (Mean ± SEM: type I, 40.4 μm ± 0.4 μm versus 37.2 μm ± 0.4 μm ; type IIA, 39.1 μm ± 0.3 μm versus 34.3 μm ± 0.3 μm; and type IIB, 45.1 μm ± 0.4 μm versus 45 μm ± 0 .4 μm). The two way ANOVA Bonferroni's multiple comparison test $p > 0.05$. **g,** The variance coefficient (VC) of the muscle fiber diameter, calculated as 1000 x SD / Mean, in both groups are shown in bar-dot plot (Mean ± SEM: type I, 182.7 ± 8.4 vs 204.1 ± 8.5; type IIA, 189.9 ± 7.9 versus 187.2 ±

12.6; and type IIB 269.6 ± 14.8 versus 264.3 ± 15.6). The bar and error bar in plots represent Mean and SEM, respectively. Scale bars = 100 μm (insets 500 μm).

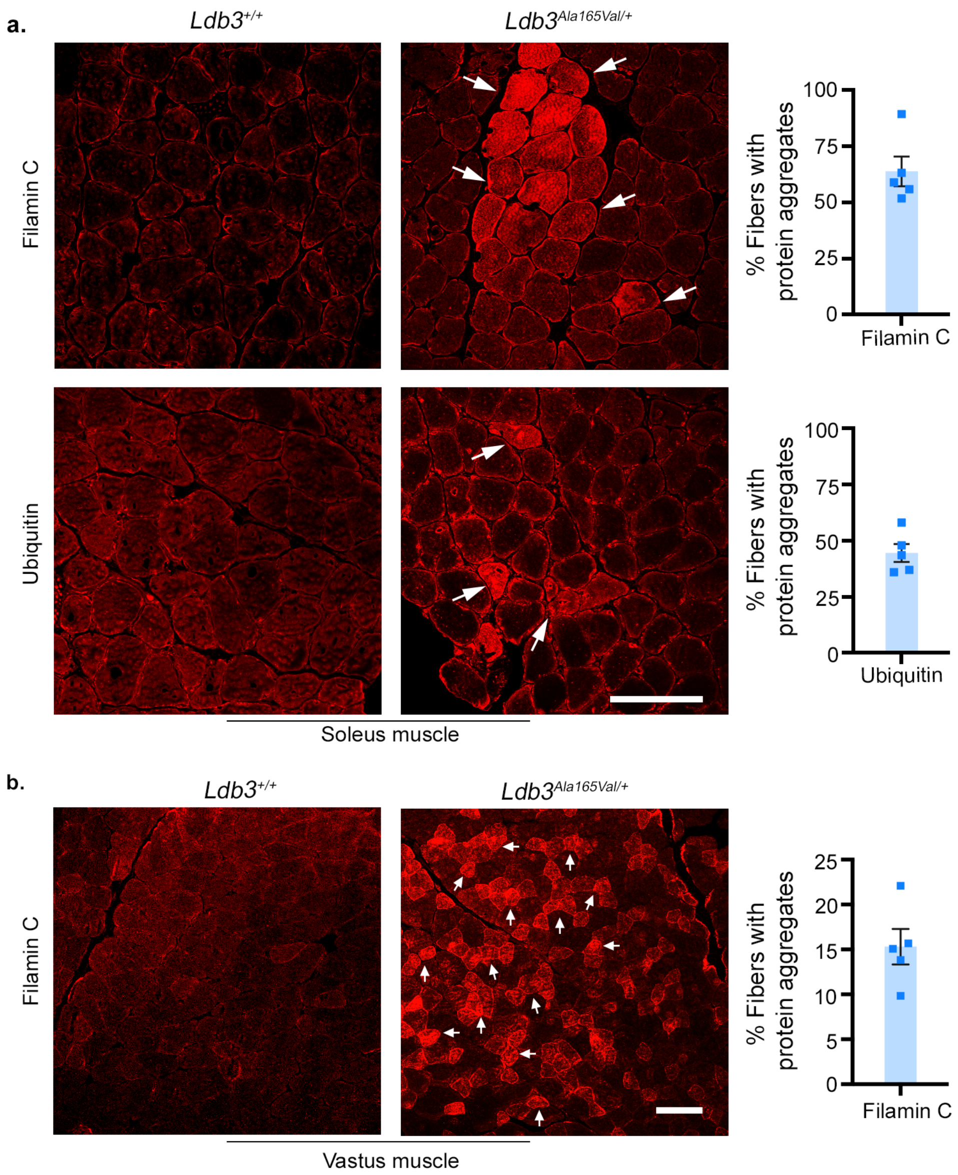

Supplementary Fig. 3: Quantitation of percentage skeletal muscle fibers with sarcoplasmic filamin C and ubiquitin accumulations in *Ldb3$^{Ala165Val/+}$* mice

a, Representative immunofluorescence images of the soleus muscle transverse section obtained from 8 month old *Ldb3$^{Ala165Val/+}$* mice and *Ldb3$^{+/+}$* mice (n = 5 each) stained with filamin C and ubiquitin antibodies, which were used for quantitation of percentage fibers with protein aggregates. Arrows show examples of counted abnormal fibers containing protein accumulations in mutant mice. Note focal cluster of abnormal fibers is

surrounded by fibers without any aggregates. Bar-dot plots show quantitation of percentage muscle fibers containing filamin C and ubiquitin accumulations in the soleus muscle of mutant mice. **b,** Representative immunofluorescence images of the vastus muscle transverse section obtained from 6 month old *Ldb3*$^{Ala165Val/+}$ mice and *Ldb3*$^{+/+}$ mice (n = 5 each) stained with filamin C antibody, which were used for quantitation of percent fibers with protein aggregates. Bar-dot plot shows quantitation of percentage muscle fibers containing filamin C accumulations in the vastus muscle of mutant mice. Muscle fibers with protein aggregates are not seen in the soleus and vastus muscles of *Ldb3*$^{+/+}$ mice. The data represent a mean of 498 fibers and 623 fibers per muscle, for the soleus and vastus muscles, respectively. The bar and error bar in plots represent Mean and SEM, respectively. Scale bars = 100 μm.

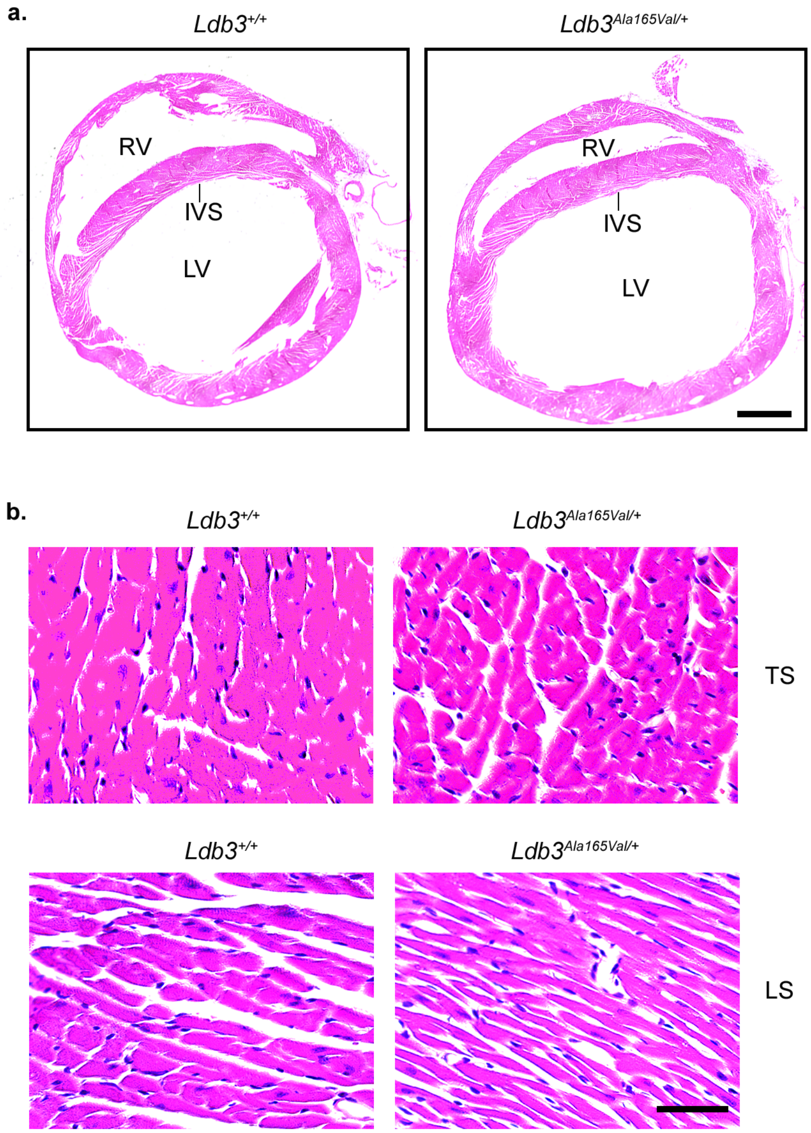

Supplementary Fig. 4: Heart histology of *Ldb3*$^{Ala165Val/+}$ mice

Representative hematoxylin and eosin stained histological sections of heart showing normal morphology in 9 month old *Ldb3*$^{Ala165Val/+}$ mice (n = 3) and *Ldb3*$^{+/+}$ littermates (n = 3). **a,** Low magnification (scale bar = 1mm) of digitally stitched images, showing the interventricular septum (IVS) and ventricular walls (RV, right ventricle; LV, left ventricle). **b,** Transverse section (TS) and longitudinal section (LS) of the left ventricular myocardium at high magnification (scale bar = 50 μm).

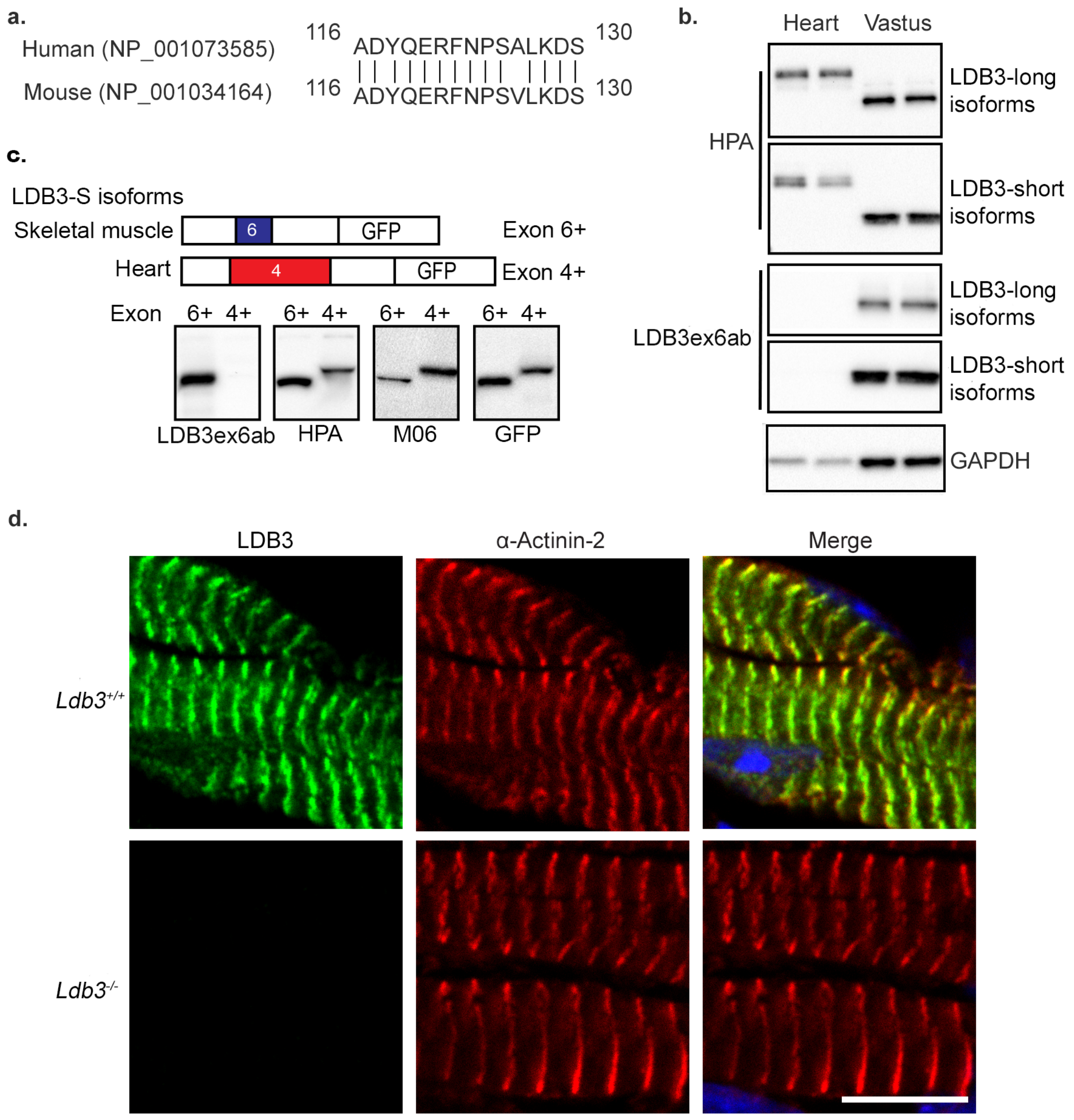

Supplementary Fig. 5: Validation of custom-generated LDB3ex6Ab antibody.

a, Epitope for custom-generated rabbit LDB3ex6Ab polyclonal antibody is shown in the human and mouse LDB3 proteins. Amino acids are numbered according to the NCBI reference protein sequence IDs shown in parenthesis. As shown, the LDB3 exon 6 encoded peptide is identical between human and mouse proteins except for a single residue. **b,** Representative immunoblot shows detection of LDB3 longer and shorter

isoforms in the heart and skeletal muscle (vastus) of wildtype mice using different LDB3 antibodies. In mice, the isoforms that contain exon 6 are predominantly expressed in skeletal muscle and the isoforms with exon 4 are predominantly expressed in heart[9]. Accordingly, LDB3ex6ab antibody detects LDB3 isoforms in skeletal muscle but not heart. In contrast, a commercially available antibody (HPA048955; Atlas Antibodies, Bromma, Sweden) detects LDB3 isoforms in both tissues. GAPDH antibody was used as a loading control. **c,** Diagram shows GFP-tagged human skeletal muscle LDB3-S isoform with exon 6 and GFP-tagged human cardiac LDB3-S isoform with exon 4 (NM_001080116 and NM_001080115, respectively), which were expressed in COS7 cells for LDB3ex6ab antibody validation assay. Representative immunoblots for detection of the GFP-tagged LDB3-S isoforms in COS7 cell lysates using different antibodies are shown below. LDB3ex6ab antibody specifically detects the skeletal muscle isoform (exon 6+), whereas commercially available HPA048955 and M-06 (Abnova, Taipei, Taiwan) antibodies show reactivity with both cardiac and skeletal muscle isoforms. See Supplementary Table 3 for antibody details. GFP antibody was used as a control. **d,** Representative immunofluorescence on the vastus muscle longitudinal sections of 18 day old *Ldb3*$^{+/+}$ embryo (n = 3) shows LDB3ex6ab antibody signal (green) co-localized with α-actinin-2 signal (red) at the Z-discs of muscle fibers. In contrast, the LDB3ex6ab antibody signal is absent from α-actinin-2 stained Z-discs in muscle fibers of age-matched *Ldb3*$^{-/-}$ embryo (n = 3). Note: *Ldb3*$^{-/-}$ mice died immediately after birth or were stillborn, as previously published[11]. Scale bar = 50 µm.

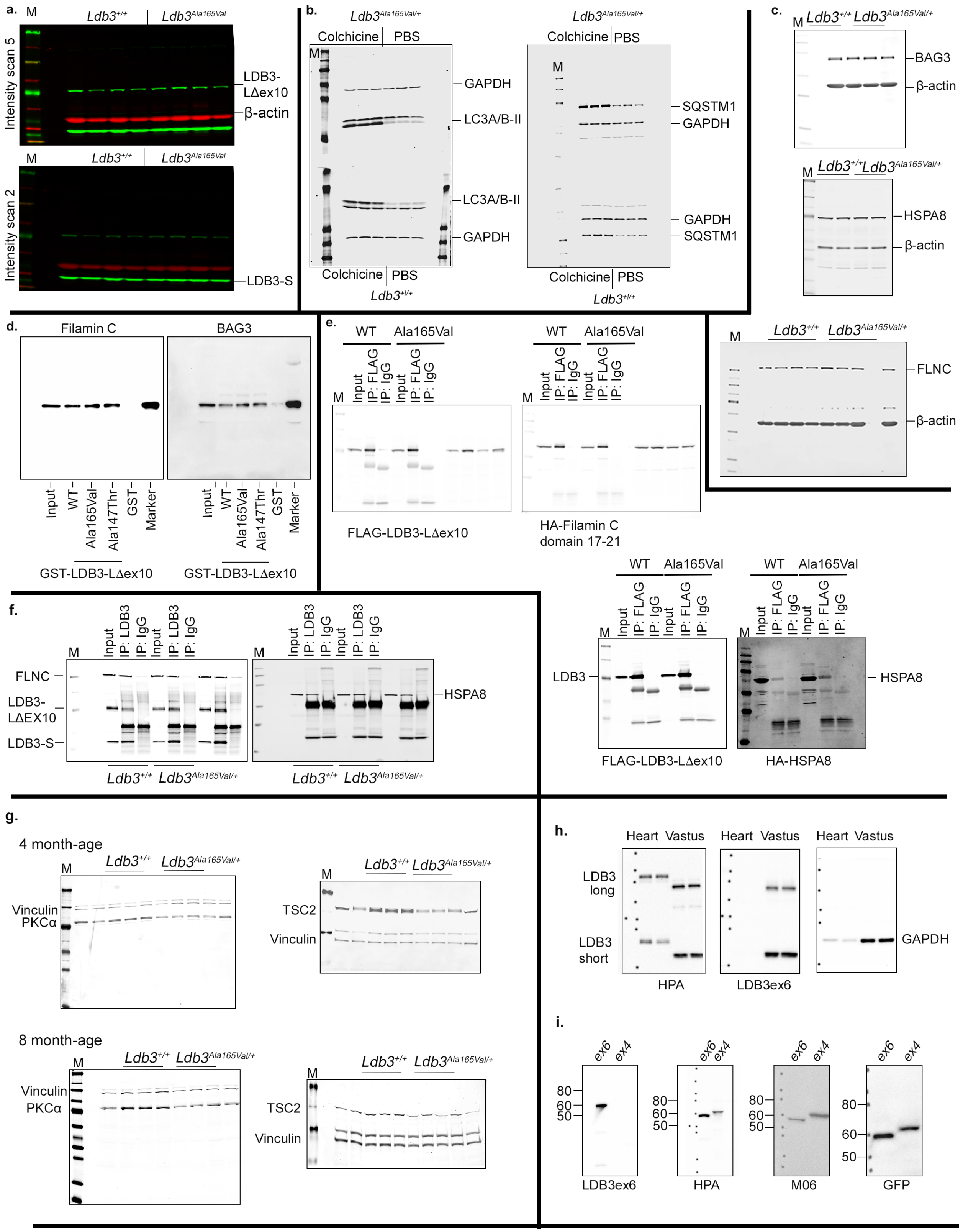
a.
M
Ldb3$^{+/+}$
Ldb3^{Ala165Val}
Intensity scan 5
LDB3-LΔex10
β-actin
Intensity scan 2
LDB3-S
b.
Ldb3$^{Ala165Val/+}$
Colchicine
PBS
M
GAPDH
LC3A/B-II
SQSTM1
Ldb3$^{+/+}$
c.
BAG3
HSPA8
d.
Filamin C
Input
WT
Ala165Val
Ala147Thr
GST
Marker
GST-LDB3-LΔex10
e.
IP: FLAG
IP: IgG
FLAG-LDB3-LΔex10
HA-Filamin C domain 17-21
FLNC
f.
IP: LDB3
LDB3-LΔEX10
LDB3
HA-HSPA8
g.
4 month-age
Vinculin
PKCα
TSC2
8 month-age
h.
Heart
Vastus
LDB3 long
LDB3 short
HPA
LDB3ex6
i.
ex6
ex4
80
60
50
M06
GFP

Supplementary Fig. 6: Full-length Western blot images

a, Full-length blot images for figure 1c; **b,** Full-length blot images for figure 2g; **c,** Full-length blot images for figure 5a; **d,** Full-length blot images for figure 5d; **e,** Full-length blot images for figure 5e; **f,** Full-length blot images for figure 5f; **g,** Full-length blot images for figure 6c and 6f; **h-i,** Full-length blot images for Supplementary figure 5b-c. M = marker lane.

Supplementary Table 1: List of LDB3 putative interactors, n ≥ 3 prey clones, encoding peptides in-frame on a sense strand, identified with a strong confidence in a yeast two-hybrid screen of a human skeletal muscle cDNA library.

cDNA	Accession (RefSeq)	Gene ID (NCBI)	Start[1]	End[1]	Known function; Location
Filamin C	NM_001127487.1	2318	5436	7080	Mechanosensor; Z-disc
HSPA8	NM_006597.5	3312	789	1497	CASA chaperone; Z-disc
HSPA5	NM_005347.4	3309	636	1560	Chaperone; ER[2]
Prohibitin 2	NM_001144831.1	11331	243	983	Mitophagy receptor; mitochondria
Ribophorin II	NM_001135771.1	6185	1071	1542	Ribosome binding; ER[2]
SIAH1	NM_003031.3	6477	390	1179	E3 ubiquitin ligase

Bait: Human LDB3 amino acid # 221 - 320 (NM_001080114; N-LexA-DBD-bait-C fusion)

Prey: Human adult + fetal skeletal muscle cDNA library (N-GAL4-AD-prey-C fusion)

[1]Maximum limit for the coverage in base pair shown.

[2] endoplasmic reticulum.

Supplementary Table 2: List of proteins with a significant fold-change (≥ 1.5; corrected p ≤ 0.05) measured by Reverse Phase Protein Array in the vastus muscle of *Ldb3*$^{Ala165Vall/+}$ mice relative to their *Ldb3*$^{+/+}$ littermates (n = 5 mice per each group, total 20 mice)

**Protein**	**Uniprot ID**	***Ldb3***$^{+/+}$ **(Log$_2$mean)**	***Ldb3***$^{Ala165Val/+}$ **(Log$_2$mean)**	**Fold change**	**Known function**
a. Age: 4 months					
PKCα	P20444	0.52	-0.28	-1.7	Protein stability
TSC2[1]	Q61037	0.22	-0.36	-1.5	mTOR regulator
Rab25	Q9WTL2	0.25	-0.34	-1.5	Ras oncogene family
PMS2	P54279	0.60	-0.41	-2.0	DNA mismatch Repair
b. Age: 8 months					
PKCα	P20444	0.46	-0.40	-1.8	Protein stability
GDH1	P26443	0.52	-0.39	-1.9	Mitochondrial matrix enzyme
PMS2	P54279	0.51	-0.32	-1.8	DNA mismatch Repair

[1] TSC2 levels showed a downward trend (fold change -1.4; corrected p = 0.07) at age 8 months

Supplementary Table 3: List of primary antibodies used in this study.

Protein	Species	Dilution	Application[1]	Company	Catalog Number	Validation/ Reference[2]
α-Actinin-2	Mouse	1:50	IF	Sigma	EA53-A7811	20858595
	Rabbit	1:1000	WB	Abcam	ab72592	26571398
αB-crystallin	Rabbit	1:500; 1:1000	IF; WB	Abcam	ab13497	28452077
BAG3	Rabbit	1:400	IF	Abcam	ab47124	22961544
		1:8000	WB	Proteintech	10599-1-AP	20060297
β-actin	Mouse	1:6000	WB	Abcam	ab6276	29295976
Desmin	Rabbit	1:400; 1:2000	IF; WB	Abcam	ab32362	27749823
Digoxigenin-AP	Sheep	1:7000	Southern blot	Roche Diagnostic	11093274910	25220466
Filamin C	Rabbit	1:50; 1:500	IF; WB	Novus Biologicals	NBP1-89300	25351925
FLAG tag	Mouse	3.3 µg; 1:15,000	IP; WB	Sigma-Aldrich	F1804	24668811
GAPDH	Mouse	1:6000	WB	Proteintech	60004-1-Ig	29657030
	Rabbit	1:12,000	WB		10494-1-AP	29311302
GFP	Mouse	1:1000	WB	Life Technologies	MA5-15256	28831037
Hemagglutinin tag	Rabbit	1:25,000	WB	Abcam	ab9110	28864826
HSPA8	Mouse	1:50; 1:2000	IF; WB	Santa Cruz Biotech	sc-7298	27650854
HSPB8	Rabbit	1:50; 1:500	IF; WB	Abcam	ab151552	28915917
LC3A/B[3]	Rabbit	1:200	WB	Cell Signaling	12741	28051178
LDB3	Mouse	1:1000; 1:10,000; 3.3 µg	IF; WB; IP	Abnova	M06-H00011155	24668811
	Rabbit	1:1000	IF; WB	Atlas antibodies	HPA048955	26109061
		1:1000; 1:10,000	IF; WB	Present study; see Supplementary Fig. 5		
MHC IIA; IIB; IIX	Mouse	1:50	IF	Developmental Studies Hybridoma Bank (DSHB)	SC-71; BF-F3; 6H1	31604915
MHC I	Mouse	1:25	IF	DSHB	BA-F8	31604915
Myotilin	Rabbit	1:300; 1:2000	IF; WB	Abcam	ab68915	24668811
PKCα	Rabbit	1:100	WB	Cell Signaling	2056	28464351
Polyubiquitin	Rabbit	1:400	IF	Abcam	ab7780	22961544
SQSTM1 / p62	Mouse	1:5000	WB	Abcam	ab56416	27349908
TSC2	Rabbit	1:500	WB	Cell Signaling	D93F2-4308	28289099
Vinculin	Mouse	1:20000	WB	Thermo-Fisher Scientific	VLN01-MS-1209	31723142

[1]IF: immunofluorescence; WB: Western blotting; IP: immunoprecipitation; [2]PubMed ID; [3]the affinity of this antibody for LC3-I and LC3-II has not been directly compared, as per the manufacturer.

Supplementary Table 4: List of primers and probes used in this study.

Target	Primers and probes (5' to 3')	Location	Assay
FLNC cDNA	AT*GAATTC*ATGACGGCAACCACATCCC[1]<br>AT*GTCGAC*TAGGTGACAGTGAGACGGCG[1]	Exon 32<br>Exon 42	PCR Cloning
HSPA8 cDNA	ACGTAT*GTCGAC*CTAGCCCTTGTCATTAGTGATAGTAATCTTG[1]<br>ATGCACT*CTCGAG*CTAATCAACCTCTTCAATGGTGGGCC[1]	Exon 2<br>Exon 9	
Mouse genomic DNA<br><br>*Ldb3* floxAla165Val/+<br>Ala165Val/+<br>+/+	cactgagactggagttgctgtaagag<br>ccacacagcagctcgctactg	Intron 3<br>Intron 4	Targeted gene sequencing
	gtctgttctcatccctgggttactgg<br>CGCGATGGCGTCCATGATAG[2]	Intron 4<br>Exon 6	
	acctctgctccgagtctcc<br>CACCAAAGAACGGAGCCGGTTG	Intron 5<br>PGK-neo[3]	
	GCTCTATGGCTTCTGAGG<br>cttgctgctggaagatgc	PGK-neo[3]<br>Intron 7	
	tccgtccgtccgtcttgtcatc<br>gagttgagagggcacgatgtagttg	Intron 7<br>Intron 8	
	tctctgctcccatcttctcc<br>aagtagtgcctttccgagacc	Intron 6<br>Intron 6	Genotyping
	tgtgtctgcttctccatcgt<br>tacCCACTGAAGTCACTGCC	Intron 5<br>Exon 6-Intron 6	PCR; Sanger sequencing
	GTACAACACCCCGATCAGCAT<br>ggcgcttacCCACTGAAGTC	Exon 6<br>Exon 6-Intron 6	SNP genotyping
	VIC-CACAGGATG**C**TATCA[4]<br>FAM-CACAGGATG**T**TATCATG[4]	Exon 6	
Mouse cDNA<br>*Ldb3* Ala165Val/+	AGGAAGCCACCGCTGTCAG<br>AACCTTTCCCTTGACCTTCGC	Exon 1<br>Exons 9-10[5]	PCR; Sanger sequencing
	GCCTATTCCCATCTCCACGACAGC<br>CAGGATGCGGAAGGAGCGAGAC	Exon 3<br>Exon 8	
	AATGCTGACTACCAGGAACGC<br>CACATGCCAGATCTGTGTGG	Exon 5-6<br>Exon 10[5]	
	GGAAGATGAGGCTGATGAGTGG<br>CCATGCATTCTTCCTGAGGTTAGC	Exon 9<br>Exon 17	
	CTCACCTGCCCCAAACTATACC	Exon 13	
	ATCAGTAGCAGGAGCAGCC	Exon 17	

[1] Recognition sequences for restriction enzymes are italicized and underlined.
[2] Whereas the underlined G is at the mutation site, this primer was not allele-specific.
[3] PGK-neo reporter was inserted in intron 6 of $Ldb3^{floxA165V}$ allele.
[4] Allele-specific base pair in the probes are in bold and underlined.
[5] Exon 10 is present in shorter (LIM absent) but, not in longer LDB3 isoform.
