# Supplementary material for: Myopathy associated LDB3 mutation causes Z-disc disassembly and protein aggregation through PKCα and TSC2-mTOR downregulation
Source: Commun Biol. 2021 Mar 19;4:355. doi: 10.1038/s42003-021-01864-1 (PMC7979776; doi:10.1038/s42003-021-01864-1)
Supplement: Supplementary file 3 — Description of Additional Supplementary Files [file 42003_2021_1864_MOESM3_ESM.pdf]

Description of Additional Supplementary Files

File Name: Supplementary Data 1

Sheet (Fig. 1c): Immunoblotting analysis of the LDB3-LDex10 and LDB3-S protein expression relative to b-actin in the vastus muscle of 8 month old *Ldb3*$^{Ala165Val/+}$ mice and *Ldb3*$^{+/+}$ littermates (n = 4 each). Table of the two-tailed unpaired t-test comparison between groups.

File Name: Supplementary Data 2

Sheet (Fig. 1d): Maximal all paws grip force (g/g) of five pulls per mouse normalized to body weight between *Ldb3*$^{Ala165Val/+}$ and *Ldb3*$^{+/+}$ littermates at 3, 6, and 9 months of age (10 – 13 mice and 8 – 10 mice, respectively). Table of the two way ANOVA Bonferroni's multiple comparisons test between groups.

File Name: Supplementary Data 3

Sheet (Fig. 1e): Maximal isometric force (mN/mm^2) generated by the extensor digitorum longus (EDL) muscle of 6 month old *Ldb3*$^{Ala165Val/+}$ mice (n =4) and *Ldb3*$^{+/+}$ littermates (n = 3) against stimulation frequencies (Hz). Table of the two way ANOVA Bonferroni's multiple comparisons test between groups.

File Name: Supplementary Data 4

Sheet (Fig. 1f): Maximum holding impulse (N*s) in the four limb wire grid holding test in 9 month old male *Ldb3*$^{Ala165Val/+}$ mice (n=9) and *Ldb3*$^{+/+}$ littermates (n = 7). Table of the two-tailed unpaired t-test comparison between groups.

File Name: Supplementary Data 5

Sheet (Fig. 2h-i): h, Immunoblotting analysis of LC3A/B-II and sequestosome-1 (p62) protein levels, relative to GAPDH, in the vastus muscle of 4 month old *Ldb3*$^{Ala165Val/+}$ mice and *Ldb3*$^{+/+}$ littermates after three days of colchicine (COL) or PBS treatment (n = 3 mice per treatment group). Table of the two way ANOVA Bonferroni's multiple comparisons between groups. **i,** LC3A/B-II and sequestosome-1 (p62) flux [ΔCOL – PBS] in *Ldb3*$^{Ala165Val/+}$ mice and *Ldb3*$^{+/+}$ littermates (n = 3 mice per treatment group). Table of the two-tailed unpaired t-test comparison between groups.

File Name: Supplementary Data 6

Sheet (Fig. 6a): Log transformed (base 2), median centered, and variance-stabilized (median absolute deviation) raw abundances detected per sample of 4 months old *Ldb3*$^{Ala165Val/+}$ mice (n = 5) and *Ldb3*$^{+/+}$ littermates (n = 5) in RPPA. An absolute difference of means ≥ 1.5x and a corrected p ≤ 0.05 (Welch modified t-test under Benjamini-Hochberg false discovery rate multiple comparison correction) were considered as differentially expressed between the groups.

File Name: Supplementary Data 7

Sheet (Fig. 6b): Log transformed (base 2), median centered, and variance-stabilized (median absolute deviation) raw abundances detected per sample of 8 months old *Ldb3*$^{Ala165Val/+}$ mice (n = 5) and *Ldb3*$^{+/+}$ littermates (n = 5) in RPPA. An absolute difference of means ≥ 1.5x and a corrected p ≤ 0.05 (Welch modified t-test under Benjamini-Hochberg false discovery rate multiple comparison correction) were considered as differentially expressed between the groups.

File Name: Supplementary Data 8

Sheet (Fig. 6d): Immunoblotting analysis of PKCa and TSC2 protein levels relative to vinculin in the vastus muscle of 4 month old *Ldb3*$^{Ala165Val/+}$ mice (n = 5 and 4, respectively) and *Ldb3*$^{+/+}$ littermates (n = 4 and 3, respectively). Table of the two-tailed unpaired t-test comparison between groups.

File Name: Supplementary Data 9

Sheet (Fig. 6e): Immunoblotting analysis of PKCa and TSC2 protein levels relative to vinculin in the vastus muscle of 8 month old *Ldb3*$^{Ala165Val/+}$ mice (n = 4 and 5, respectively) and *Ldb3*$^{+/+}$ littermates (n = 3 and 4, respectively). Table of the two-tailed unpaired t-test comparison between groups.
