# Supplementary material for: Myopathy associated LDB3 mutation causes Z-disc disassembly and protein aggregation through PKCα and TSC2-mTOR downregulation
Source: Commun Biol. 2021 Mar 19;4:355. doi: 10.1038/s42003-021-01864-1 (PMC7979776; doi:10.1038/s42003-021-01864-1)
Supplement: Supplementary file 13 — Reporting Summary [file 42003_2021_1864_MOESM13_ESM.pdf]

nature research

Corresponding author(s): Ami Mankodi

Last updated by author(s): 01/29/2021

Reporting Summary

Nature Research wishes to improve the reproducibility of the work that we publish. This form provides structure for consistency and transparency in reporting. For further information on Nature Research policies, see our Editorial Policies and the Editorial Policy Checklist.

Statistics

For all statistical analyses, confirm that the following items are present in the figure legend, table legend, main text, or Methods section.

n/a	Confirmed	
☐	☒	The exact sample size (n) for each experimental group/condition, given as a discrete number and unit of measurement
☐	☒	A statement on whether measurements were taken from distinct samples or whether the same sample was measured repeatedly
☐	☒	The statistical test(s) used AND whether they are one- or two-sided<br>*Only common tests should be described solely by name; describe more complex techniques in the Methods section.*
☒	☐	A description of all covariates tested
☐	☒	A description of any assumptions or corrections, such as tests of normality and adjustment for multiple comparisons
☐	☒	A full description of the statistical parameters including central tendency (e.g. means) or other basic estimates (e.g. regression coefficient) AND variation (e.g. standard deviation) or associated estimates of uncertainty (e.g. confidence intervals)
☐	☒	For null hypothesis testing, the test statistic (e.g. F, t, r) with confidence intervals, effect sizes, degrees of freedom and P value noted<br>*Give P values as exact values whenever suitable.*
☒	☐	For Bayesian analysis, information on the choice of priors and Markov chain Monte Carlo settings
☒	☐	For hierarchical and complex designs, identification of the appropriate level for tests and full reporting of outcomes
☒	☐	Estimates of effect sizes (e.g. Cohen's d, Pearson's r), indicating how they were calculated

Our web collection on statistics for biologists contains articles on many of the points above.

Software and code

Policy information about availability of computer code

Data collection	QuantStudio 6 Flex Real-Time PCR System (Applied Biosystems), Agilent 2200 TapeStation using the D1000 Kit (Agilent Technologies), Illumina MiSeq, ChemiDoc imager (BioRad), Gross motor performance: RotaRod Series 8 (IITC Life Sciences, CA, USA), Grip Strength: BIOSEB grip strength meter (BIOSEB, Vitrolles, France), Four limb hangTest: NIH Instrumentation core apparatus, Leica DMI 6000 SD microscope, Confocal microscope (TCS SP5 576 II, Leica) equipped with a Plan-Apochromat 20x, 40x and 60x oil immersion objective lens , Aurora 1200A in vitro dual lever system (Aurora Scientific Inc.), Electron microscope (JEOL 200CX, Jeol, Inc.), Odyssey Imaging Systems (LI-COR Biosciences).
Data analysis	IITC Instrument Suite Monitor version 1.29, Bioseb BIO-CIS version 1.5.1.0, GraphPad prism 8, 610A DMC Version 5.300, R-package, Adobe Photoshop Creative Cloud v2017, ImageJ, Image Lab software (version 5.2, Biorad), Image Studio Lite software (LI-COR Biosciences), Array-Pro Analyzer 6.3.

For manuscripts utilizing custom algorithms or software that are central to the research but not yet described in published literature, software must be made available to editors and reviewers. We strongly encourage code deposition in a community repository (e.g. GitHub). See the Nature Research guidelines for submitting code & software for further information.

Data

Policy information about availability of data

All manuscripts must include a data availability statement. This statement should provide the following information, where applicable:

- Accession codes, unique identifiers, or web links for publicly available datasets
- A list of figures that have associated raw data
- A description of any restrictions on data availability

The authors declare that the data supporting the findings of this study are available within the publication and supplementary data files. All data of this study are available from the corresponding author upon reasonable request.

Field-specific reporting

Please select the one below that is the best fit for your research. If you are not sure, read the appropriate sections before making your selection.

☒ Life sciences ☐ Behavioural & social sciences ☐ Ecological, evolutionary & environmental sciences

For a reference copy of the document with all sections, see nature.com/documents/nr-reporting-summary-flat.pdf

Life sciences study design

All studies must disclose on these points even when the disclosure is negative.

Sample size	No sample size calculation was done. Sample sizes were guided by The TREAT-NMD SOP guidelines, which range about 6-9 animals per group.
Data exclusions	None
Replication	At-least three independent replicates for all data obtained in the study.
Randomization	Random assignment of mice for all studies.
Blinding	Investigators were blinded to genotype and to treatment assignment during data collection and analysis.

Reporting for specific materials, systems and methods

We require information from authors about some types of materials, experimental systems and methods used in many studies. Here, indicate whether each material, system or method listed is relevant to your study. If you are not sure if a list item applies to your research, read the appropriate section before selecting a response.

Materials & experimental systems

n/a	Involved in the study	
☐	☒	Antibodies
☐	☒	Eukaryotic cell lines
☒	☐	Palaeontology and archaeology
☐	☒	Animals and other organisms
☒	☐	Human research participants
☒	☐	Clinical data
☒	☐	Dual use research of concern

Methods

n/a	Involved in the study	
☒	☐	ChIP-seq
☒	☐	Flow cytometry
☒	☐	MRI-based neuroimaging

Antibodies

Antibodies used	Information for Antibody Species, Dilution, Application, Company name and Catalog Number are included in Supplementary Table 3. Alpha B Crystallin,BAG3, Beta-actin, Desmin, Digoxigenin-AP Fab Fragment, Filamin C, Flag tag, GAPDH, GFP tag, HA tag, Hsc70, Hsp22, LC3-I/-II, LDB3, myosin heavy chain (MHC) types I, IIA, IIB, and IIX (BA-F8, SC-71, BF-F3, and 6H1, respectively), Myotilin, PKC alpha, PolyUbiquitin, Sarcomeric α-Actinin, SQSTM1 / p62, Tuberin, Vinculin, Anti-Mouse IgG2b Cross-Adsorbed Alexa Fluor 488 and568, Anti-Mouse IgG (H+L) Highly Cross-Adsorbed, Anti-Rabbit IgG (H+L) Highly Cross-Adsorbed AF 488, Anti-Mouse IgG (H+L) Highly Cross-Adsorbed AF 568, Anti-Rabbit IgG (H+L) Highly Cross-Adsorbed AF 568, Anti mouse IgM – AF 568, Anti mouse CY5-IgG1, IRDye® 680RD-conjugated anti-Mouse, IRDye® 680RD-conjugated anti-Rabbit, IRDye® 800CW-conjugated anti-Mouse, IRDye® 800CW-conjugated anti-Rabbit, IRDye® 800CW-conjugated anti-Mouse, IRDye® 800CW-conjugated anti-Rabbit. A custom-made antibody against LDB3 was generated by immunizing rabbits with a peptide corresponding to amino acid residues 116 – 130 encoded by exon 6 of human LDB3 (NP_001073585; LDB3ex6ab; Supplementary Fig. 5a).
Validation	We validated the LDB3ex6ab antibody for immunoblotting and immunofluorescence using transfected COS-7 cells and tissues of wildtype and Ldb3-/- mice (Supplementary Fig. 5b-d). The antibodies used for Western blots and immunofluorescence were already validated by manufacturers in appropriate cell lines, tissues/ KO tissues and had multiple citations (Antibody catalog number and at least one key article published for candidate antibody are provided in Supplementary Table 3). The linear range for protein loading and antibody dilution curves were obtained. Appropriate secondary antibodies preabsorbed against multiple mammalian species used. No-primary-antibody controls were always used to verify specificity. Alpha B Crystallin (PMID: 28452077), BAG3 (PMID: 22961544, PMID: 20060297), Beta-actin (PMID: 29295976) , Desmin (PMID: 27749823), Digoxigenin-AP Fab Fragment (PMID: 25220466), Filamin C (PMID: 29078393), Flag tag (PMID: 24668811), GAPDH (PMID: 29657030 , PMID: 29311302), GFP tag (PMID: 28831037), HA tag (PMID: 28864826), Hsc70 (PMID: 27650854), Hsp22 (PMID: 28915917), LC3-I/-II (PMID: 28051178), LDB3 (PMID: 24668811, PMID: 26109061), MHC I, IIA; IIB; IIX (PMID: 3160491532), Myotilin (PMID: 24668811), PKC alpha (PMID: 28464351), PolyUbiquitin (PMID: 22961544), Sarcomeric α-Actinin (PMID: 20858595), SQSTM1 / p62 (PMID: 27349908), Tuberin (PMID: 28289099), Vinculin (PMID: 31723142). Anti-Mouse IgG2b Cross-Adsorbed Alexa Fluor 488 (PMID: 27019136), Anti-Mouse IgG (H+L) Highly Cross-Adsorbed (PMID: 30504831), Highly Cross-Adsorbed Anti mouse IgM – AF 568 (PMID: 29845793), Preadsorbed Anti

mouse CY5-IgG1 (PMID: 30528433), Anti-Rabbit IgG (H+L) Highly Cross-Adsorbed AF 488 (PMID: 29540680), Anti-Mouse IgG (H+L) Highly Cross-Adsorbed AF 568 (PMID: 30692273), Anti-Rabbit IgG (H+L) Highly Cross-Adsorbed AF 568 (PMID: 30692273), IRDye® 680RD-conjugated anti-Mouse (PMID: 28691711), IRDye® 680RD-conjugated anti-Rabbit (PMID: 27699224), IRDye® 800CW-conjugated anti-Mouse (PMID: 27699224), IRDye® 800CW-conjugated anti-Rabbit (PMID: 28691711), IRDye® 800CW-conjugated anti-Mouse (PMID: 27699224), IRDye® 800CW-conjugated anti-Rabbit (PMID: 28691711).

Eukaryotic cell lines

Policy information about cell lines

Cell line source(s)	COS-7 cells were obtained from ATCC.
Authentication	COS-7 cells obtained from ATCC were not further authenticated.
Mycoplasma contamination	COS-7 cells obtained from ATCC tested negative for mycoplasma.
Commonly misidentified lines (See ICLAC register)	N/A

Animals and other organisms

Policy information about studies involving animals; ARRIVE guidelines recommended for reporting animal research

Laboratory animals	C57BL/6N402Atm1Brd/a Ldb3tm2a(EUCOMM)Hmgu/BcmMmucd), LDB3-Ala165Val/+. The Ldb3Ala165Val/+ mice were backcrossed for at least 5 generations on C57BL/6N background. Both males and females from 2 to 12 months age were used in this study.
Wild animals	The study did not involve wild animals.
Field-collected samples	The study did not samples collected from fields.
Ethics oversight	All animal studies were authorized by the Institutional Animal Care and Use Committee of the NIH/NINDS.

Note that full information on the approval of the study protocol must also be provided in the manuscript.
